# Phage K *gp102* Drives Temperature-Sensitive Antibacterial Activity on USA300 MRSA

**DOI:** 10.3390/v15010017

**Published:** 2022-12-21

**Authors:** Susan M. Lehman, Rohit Kongari, Adam M. Glass, Matthew Koert, Melissa D. Ray, Roger D. Plaut, Scott Stibitz

**Affiliations:** Center for Biologics Evaluation and Research, US Food and Drug Administration, Silver Spring, MD 20993, USA

**Keywords:** bacteriophage, *Staphylococcus aureus*, temperature-sensitive, phage k, USA300, phage-host interactions

## Abstract

There is widespread interest in using obligately lytic bacteriophages (“phages”) to treat human bacterial infections. Among *Staphylococcus aureus* infections, the USA300 lineage is a frequent cause of invasive disease. We observed that phage K, a model *S. aureus* myophage, exhibits temperature-sensitive growth on USA300 strains, with the wild-type phage providing poorer growth suppression in broth and forming smaller and fainter plaques at 37 °C vs. 30 °C. We isolated 65 mutants of phage K that had improved plaquing characteristics at 37 °C when compared to the parental phage. In all 65 mutants, this phenotype was attributable to loss-of-function (LoF) mutations in *gp102*, which encodes a protein of unknown function that has homologs only among the *Herelleviridae* (SPO1-like myophages infecting gram-positive bacteria). Additional experiments with representative mutants consistently showed that the temperature-sensitive plaque phenotype was specific to USA300 MRSA strains and that Gp102 disruption was correlated with improved suppression of bacterial growth in broth and improved antibacterial activity in a mouse model of upper respiratory tract infection. The same genotype and in vitro phenotypes could be replicated in close relatives of phage K. Gp102 disruption did not have a detectable effect on adsorption but did delay cell culture lysis relative to wild-type under permissive infection conditions, suggesting that *gp102* conservation might be maintained by selective pressure for more rapid replication. Expression of *gp102* on a plasmid was toxic to both an MSSA and a USA300 MRSA strain. Molecular modeling predicts a protein with two helix-turn-helix domains that displays some similarity to DNA-binding proteins such as transcription factors. While its function remains unclear, *gp102* is a conserved gene that is important to the infection process of *Kayvirus* phages, and it appears that the manner in which USA300 strains defend against them at 37 °C can be overcome by *gp102* LoF mutations.

## 1. Introduction

There is widespread interest in using obligately lytic (i.e., non-temperate or non-lysogenic) bacteriophages to treat human bacterial infections, particularly infections that do not readily respond to antibiotics due to either inherent resistance, acquired resistance genes, or other factors that facilitate bacterial survival such as biofilms or high bacterial inoculum [1,2]. Much of this interest is focused on the six ESKAPE pathogens (*Enterococcus faecium*, *Staphylococcus aureus*, *Klebsiella pneumoniae*, *Acinetobacter baumannii*, *Pseudomonas aeruginosa*, and *Enterobacter* spp.), all of which exhibit concerning levels of antibiotic resistance around the world. Our lab is investigating the genetic factors that affect interactions between *S. aureus* and its phages.

Among the lytic *S. aureus* phages, the myophages with dsDNA genomes ca. 140 kb are frequently considered for therapeutic applications (reviewed by [1]). These phages currently include two genera, *Kayvirus* and *Silviavirus* [3]. Phage K is widely treated as a model phage for the *Kayvirus* genus. One notable phenotype of phage K is that, while it forms clear, 1–2 mm diameter plaques on many *S. aureus* strains when grown at 30 °C, plaque formation can be much poorer at 37 °C. When incubated at 37 °C on some *S. aureus* strains, phage K forms small, faint plaques whose visibility can be media-dependent. In the course of two separate projects, we collected a large number of phage K mutants that form larger, clearer plaques than the parent phage when plated at 37 °C on two USA300 strains. Upon sequencing the mutants, we discovered that all of them carried apparent loss-of-function (LoF) mutations in the same gene of unknown function, CPT_phageK_gp102 (per NCBI RefSeq genome NC_005880).

Even in well-studied phages such as K, the functions of more than half of the predicted genes are often unknown [4,5]. As the ease, affordability, and performance of genomic sequencing have improved, the pace of phage gene discovery has exceeded the pace of functional characterization. Some progress has been made by employing screens for protein–protein interactions [6,7,8], for the effects of phage gene expression on host cells [8,9], or for essential phage genes using classical gene deletion methods [10]. However, all of these screening methods still require extensive gene-specific follow-up. The prevalence of phage ORFans, genes of unknown function that are only found in a few closely related phages [11], also means that many results of such functional studies are not broadly generalizable.

Our serendipitous discovery of a genotype–phenotype association involving phage K *gp102* led us to study this gene in detail. We tested the effects of selected *gp102* mutations on the phage’s ability to suppress *S. aureus* growth in broth and to reduce *S. aureus* counts in the airways of chronically colonized mice. Overall, we found that loss of *gp102* function was correlated with increased phage K activity in broth and in vivo, and we replicated the in vivo findings in four closely related phages. Phage adsorption was not affected. However, under permissive infection conditions, phage K with wild-type *gp102* had a growth advantage, lysing the host faster; *gp102* loss-of-function mutations were only an advantage under the non-permissive condition in which wild-type phage K was relatively ineffective. Separately, RNA-Seq experiments showed that *gp102* transcription occurs early in phage infection, and molecular modeling suggested structural similarities to proteins involved in DNA-binding and transcriptional regulation. These structural comparisons are preliminary, but they suggest hypotheses that we plan to test in future studies. While we are currently unable to propose a molecular model of *gp102* action, it is clear that this gene is broadly conserved among the K-like phages. We hypothesize that conservation of functional *gp102* might be maintained by selection for more rapid cell lysis and that loss-of-function mutants might have an advantage only on USA300 strains.

## 2. Methods

### 2.1. Bacteria and Bacteriophages

Phage K and its growth host, ATCC 19685 were originally obtained from the American Type Culture Collection. Phage 812 and its growth host, HER#1475, were obtained from the Felix d’Herelle Reference Centre in Laval, Quebec. Three additional phages related to K were isolated from commercially obtained samples of Eliava BioPreparations products Intesti, Fersisi, and Staphylococcal Bacteriophage. Isolates were subjected to at least three rounds of single-plaque purification, followed by propagation on *S. aureus* ATCC 19685.

*S. aureus* strains were obtained from a variety of sources (Table S2). Derivatives of *S. aureus* LAC containing clean, in-frame deletions of *potA*, *potB*, *potC*, or *potD* were generated by allelic exchange as previously described [12,13] and confirmed by PCR and sequencing. Those genes were targeted after a screen of the Nebraska *S. aureus* transposon library indicated their involvement in phage K resistance. Plasmid constructs were prepared by GenScript USA (Piscataway, NJ, USA).

### 2.2. Phage Production and Quantification

Plaque assays were conducted by combining 100 µL of overnight bacterial broth culture with molten overlay agar at 50 °C and pouring over agar plates (3 mL overlay for 90 mm round plates or 6 mL overlay for 100 mm square plates). For full-plate titers, an aliquot of phage dilution was included in the molten overlay before pouring. For spot titers, aliquots of multiple phage dilutions were dropped on top of the solidified agar overlay. Phage titers were calculated based on the volume and dilution factor of the aliquot that produced discrete, countable plaques.

Plate lysates were produced using the method described for full-plate titers, except using the phage dilution that generated near-confluent or just-confluent lysis. Phages were then collected by diffusion into broth or SM buffer and passed through a 0.22 µm filter to remove bacterial and agar debris. Broth lysates were produced by inoculating sterile broth with ATCC 19685 (GHT mutants; growth at high temperature) or the LAC∆*pot* strain on which they were initially isolated (GHTP mutants: growth at high temperature on pot gene deletions) and incubating at 30 °C, 200 rpm. When cell density reached OD_600_ of approximately 0.3, phage was added to achieve an approximate initial PFU:CFU ratio between 0.1 and 1. After the culture visibly cleared, between 5 h and 20 h later, lysates were harvested by centrifugation and 0.22 µm filtration. All lysates were stored at 4 °C.

### 2.3. Isolation of Phage K Mutants

The GHT mutants were isolated by plating phage K on NRS384 using tryptic soy broth (TSB), adding 1.5% agar for plates and 0.5% agar with 5 mM calcium chloride for overlays. Well-isolated clear plaques that formed after overnight incubation at 37 °C were picked into broth and single-plaque purified twice more.

To isolate the GHTP mutants, phage K was plated on ATCC 19685 using heart infusion broth (HIB), adding 1.5% agar for plates and 0.7% agar for overlays. Six well-isolated plaques were picked into 0.5 mL SM buffer to create genetically independent parent stocks (named K1 through K6) for subsequent mutant isolation. Serial dilutions of each parent stock were plated on LAC∆*potA*, LAC∆*potB*, LAC∆*potC*, and LAC∆*potD*, at 37 °C. From each combination of phage K parent stock and plating host, up to three clear plaques (similar to those produced by K on ATCC 19685) were picked into SM buffer. Each mutant phage isolate was plaque-purified a total of three times before making a plate lysate and then a broth lysate. Each mutant isolate was titered on LAC and on its four *pot* gene deletion derivatives using the spot titer method. No allele specificity was observed, i.e., the plaquing phenotype of a given phage on the four *pot* mutants was the same, regardless of which *pot* gene deletion had been used to isolate it. Therefore, whole genome sequencing was carried out for one GHTP mutant per combination of phage K parent stock and plating host (*n* = 24).

### 2.4. Frequency of gp102 Mutations

Phage K was plated on ATCC 19685 using HIB agar. Four well-isolated plaques were picked into 0.6 mL SM buffer to create independent parent stocks as biological replicates. Using the full-plate titer method, each stock was plated on ATCC 19685, LAC, LAC∆*potA*, LAC∆*potB*, LAC∆*potC*, and LAC∆*potD*, at 37 °C. The frequency of plaques resembling those produced by K on ATCC 19685 was calculated as a proportion of each stock’s titer on ATCC 19685.

### 2.5. Phage Sequencing and Analysis

Four GHT mutants and twenty-four GHTP mutants were subjected to whole genome sequencing. Phage genomic DNA was isolated by treating filtered broth lysates with nucleases to remove bacterial DNA, followed by proteinase K treatment and isolation of the phage DNA using either the Promega Wizard kit (Promega Corporation, Madison, WI, USA; used for GHT mutants) or organic extraction and salt-ethanol precipitation (for GHTP mutants). NexteraFlex genomic libraries were prepared using 5 cycles of PCR and sequenced using Illumina MiSeq (PE150 reads). Quality-filtered reads were assembled to the phage K reference sequence (NC_005880) using Geneious. Base variants having > 10% frequency were called using the native Geneious variant caller. Since LTR boundaries are prone to mapping errors, low-frequency (<30%) and/or high strand-bias (>75%) variants in these regions were discarded.

For the remaining GHTP mutants, *gp102* and its upstream region were amplified by PCR and sequenced by Eurofins. PCR reactions used Phusion polymerase (New England Biolabs, Ipswich, MA, USA) with an annealing temperature of 64 °C for primers 5′-GACCAAGAAAAAGATGTTGC-3′ (gp102A) and 5′- CTACTTCAGGTTCTTCAGGAG-3′ (gp102D). The same primers were used for amplicon sequencing following purification using the QIAquick PCR purification kit (QIAGEN Sciences, Germantown, MD, USA).

Whole-genome sequencing of phages and *gp102* sequencing of their mutants were conducted as described above, except that PCR-free genomic libraries were used when sufficient DNA was available. Direct terminal repeats were identified from either the MiSeq read data (if a PCR-free library was used) or based on sequence similarity. To support visual comparison of these genomes with phage K, the Geneious live annotate function was used to transfer gene annotations from NC_005880 if there was a minimum 70% nucleotide similarity.

### 2.6. Bacterial Sequencing and Analysis

Table S2 lists the source of genome information for most *S. aureus* strain sequences. In addition, we sequenced strains for which no sequence data were publicly available or when we wished to confirm the sequence of our local stock. Briefly, we purified genomic DNA via lysis with lysostaphin (20 U per 100 μL of washed and resuspended cells) and organic extraction (25:24:1 buffered phenol:chloroform: isoamyl alcohol, followed by 24:1 chloroform:isoamyl alcohol) [14]. PacBio sequencing and genome assembly was conducted by the University of Maryland School of Medicine Institute for Genome Sciences. Strain characteristics were evaluated using MLST v1.6, spaTyper v1.0, SCCmecFinder v1.2, and VirulenceFinder 2.0 as hosted by the Center for Genomic Epidemiology (https://cge.food.dtu.dk/). We defined USA300 as strains that are known to have either (i) the USA300 PFGE profile or (ii) all of the following genetic traits: ST8, SCCmec type IVa, and positive for the Panton–Valentine leukocidin genes (PVL+) [15,16,17]. In all but one case, these strains were also Ridom spa type t008, which is tightly, but imperfectly, correlated with the USA300 lineage [15,16].

### 2.7. Bacterial Growth Suppression in Broth

Growth suppression assays were run in 96-well, U-bottom, non-tissue culture-treated plates. Each well initially contained approximately 3 × 10^6^ CFU and 2 × 10^5^ PFU. Growth curves were run in a BioTek Cytation3 plate reader using a discontinuous kinetic program in which OD_600_ readings were collected every 15 min for 24 h, while plates were maintained at either 30 or 37 °C with orbital shaking between OD readings.

### 2.8. Bacterial Killing in Broth

Bacterial killing assays were run in 250 mL Erlenmeyer flasks. Twelve flasks (six per strain) were inoculated with 100 μL of a 16 h culture of either ATCC 19685 (MSSA) or NRS384 (USA300), then incubated with 200 rpm shaking. When the bacterial control flasks reached OD_600_ ≈ 0.3, a 1 mL sample was removed from each flask for precise measurements of both OD_600_ and CFU counts prior to phage addition. For five flasks per *S. aureus* strain, 1 mL of 5 × 10^8^ PFU/mL phage was added to each flask, for a target initial PFU:CFU ratio of 0.1. The flasks were swirled to mix and a 1 mL sample was removed from each, passed through a 0.22 μm syringe filter, and stored at 4 °C for later PFU quantification. Every hour after phage addition for 5–6 h, a 1 mL sample was collected from each flask for OD_600_ measurement followed by filtration and storage for PFU counts; a final sample was collected approximately 21 h after phage addition. In total, the experiment was run twice each at 30 °C and 37 °C.

### 2.9. Molecular Modeling

HHPred (https://toolkit.tuebingen.mpg.de/tools/hhpred; version 14 July 2022; [18]) was run with default parameters, using the 147 amino acid sequence of phage K Gp102 as query. The full model of AlphaFold v2.0.1 was downloaded (https://github.com/deepmind/alphafold/) and run on a local HPC cluster [19]. The resulting PDB files were used as query structures in DALI [20]. Searches were conducted against the 22 March 2022 version of the PDB90 database, which is a non-redundant subset of proteins that have <90% sequence identity with each other, and against the database of *S. aureus* proteins, which is based on AlphaFold2 predictions. Results with Z scores better than 4 were manually filtered to exclude visually obvious errors in overall alignment.

### 2.10. Adsorption Assays

*S. aureus* strains were grown in 25 mL TSB at either 30 °C or 37 °C, with shaking at 200 rpm. Upon reaching an OD_600_ of 0.5–1.0, 5 mL of these log-phase bacteria were harvested by 10 min centrifugation at 4500× *g*, then resuspended in 0.9 mL of fresh TSB in microcentrifuge tubes. 100 µL of a known concentration of phages between 10^6^ and 10^7^ PFU was added to each resuspended bacteria sample and to a bacteria-free control tube. The tubes were then incubated at 30 °C or 37 °C with shaking at 120 rpm. At 5 min intervals, 200 µL samples were transferred to pre-chilled microcentrifuge tubes, briefly vortexed, and immediately centrifuged for 10 min at maximum speed at 4 °C. The number of free phages in the supernatant at each time point were quantified using the spot titer method. The percentage of free (unadsorbed) phages was calculated relative to the plaque counts obtained from a bacteria-free control tube that was processed in parallel at 0 min.

### 2.11. Animal Experiments

A murine model of upper respiratory tract decolonization was established in 8- week-old female BALB/c mice (Jackson Laboratories). Briefly, an *S. aureus* overnight culture in TSB was twice spun down and resuspended in PBS. Approximately 10^7^ CFU of *S. aureus* NRS384 (USA300 MRSA) in PBS were then delivered intranasally on Day 0. In colonization experiments, mice were euthanized 1, 2, 8, or 12 days after bacterial inoculation (*n* = 5 per time point). In treatment experiments, animals (*n* = 5 per group) were treated with either phage (10^9^–10^10^ PFU total) or saline, delivered both intranasally and intraperitoneally to each animal five times, twice each on days 7 and 8 and once on the morning of day 9; mice were sacrificed on the afternoon of day 9. For CFU and PFU counts, the turbinates, nasal vestibule, and hard palate of each mouse were excised, homogenized in PBS, and promptly dilution-plated on TSA. Little or no evidence of phage activity was observed on the CFU count plates, suggesting that ex vivo killing of bacteria by phages did not substantially impact experimental results.

### 2.12. gp102 Expression

The *gp102* open reading frame was cloned into the pT104 plasmid under the control of an arsenite-inducible promoter. The pT104 plasmid was described by Liu et al. [9] and obtained from Gail Christie. ORF synthesis, cloning, and addition of a chloramphenicol resistance marker were conducted by GenScript (Piscataway, NJ, USA). The plasmid was transformed into *S. aureus* RN4220 and NRS384 by electroporation [21]. Transformants were isolated on TSA plates containing 10 µg/mL chloramphenicol. Three transformant colonies of each strain were transferred into TSB containing 10 µg/mL chloramphenicol and grown overnight at 37 °C, 200 rpm. Serial dilutions were replica-spotted onto TSA with or without 5 µM NaAsO_2_, and incubated overnight at either 30 °C or 37 °C.

## 3. Results

### 3.1. Isolation and Identification of Phage K gp102 Mutants

**Figure 1:**
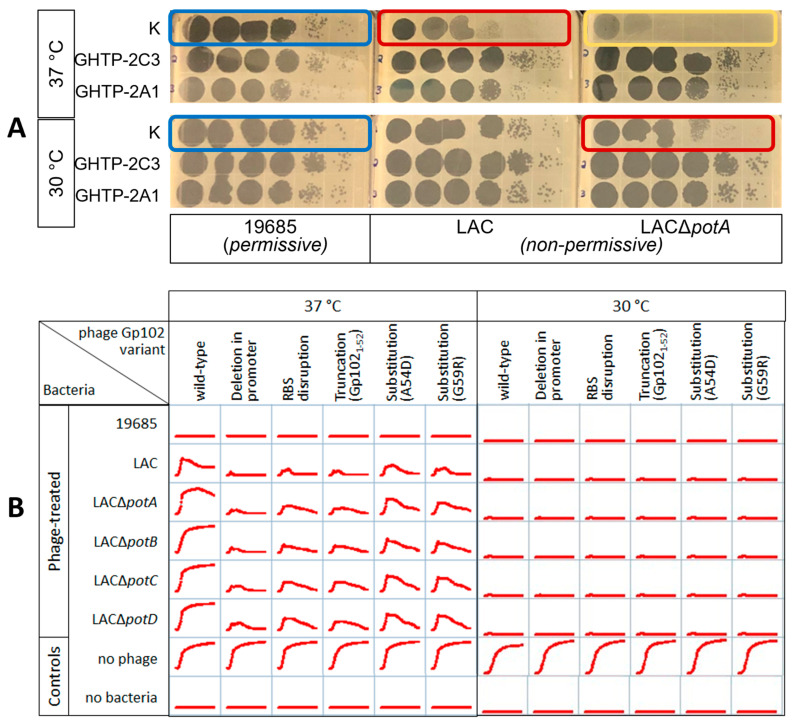
Gp102 loss-of-function mutations in phage K result in larger, clearer plaques and better growth suppression under non-permissive culture conditions (growth at 37 °C on LAC). (**A**) 10-fold dilution series of wild-type (WT) K and two *gp102* loss-of-function mutants. On the permissive host, growth of wild-type K is not temperature-sensitive (blue boxes). On the non-permissive hosts, the effects of temperature and deletion of polyamine transporter components (red boxes) are additive, further reducing plaque formation (gold box) unless *gp102* function is impaired. (**B**) 24 h bacterial growth curves in 96-well plates show the differential abilities of wild-type phage K and various types of *gp102* loss-of-function mutants to suppress growth of permissive and non-permissive *S. aureus* strains. Each well initially contained approximately 3 × 10^6^ CFU and 2 × 10^5^ PFU.

Our lab routinely grows phage K on *S. aureus* strain ATCC 19685. When plated on this and many other strains using the double agar overlay technique, phage K forms clear, 1–2 mm diameter plaques. However, when using tryptic soy agar (TSA), our phage K laboratory stock plaques on *S. aureus* strains NRS384 and LAC only when incubated at 30 °C, not 37 °C. We obtain better plaque visualization when using heart infusion agar (HIBA) instead of TSA. However, even on HIBA, phage K forms very small, faint plaques on NRS384 or LAC when incubated at 37 °C; we also confirmed an observation from our previous transposon library screening, in which the deletion of any of the pot*ABCD* genes from LAC further inhibits plaque formation (Figure 1A). Using two different selection methods, we isolated 65 clear plaques under conditions where the parental phage K did not produce clear plaques. One set of four such isolates was obtained when phage K was plated on NRS384 using TSA and 37 °C. We have called these GHT mutants (growth at high temperature). A second set of 61 isolates was obtained when phage K was plated on LACΔ*potA*, LACΔ*potB*, LACΔ*potC*, or LACΔ*potD* using HIBA and 37 °C. We have called these GHTP mutants (growth at high temperature on pot deletions).

Given the different circumstances of isolation, we were surprised that all 65 isolates contained mutations in the same gene, *gp102* (per NCBI RefSeq genome NC_005880) (Table S1). In total, the 65 isolates represent 31 distinct mutations at 29 positions within *gp102*. Many of the mutations are clearly loss-of-function mutations, such as nonsense mutations that would lead to truncated proteins or serious disruptions of the promoter or ribosome-binding sequences that would impair or abolish gene expression. Based on their similar phenotype, we infer that the missense mutations in our collection are also loss-of-function mutations, even though they are predicted to encode full-length proteins. For most of the 28 mutants subjected to whole genome sequencing, the *gp102* mutations were the only genetic differences from the parent stock; there were only four isolates in which a second mutation was observed elsewhere in the phage genome, and each was in a different gene. Given the consistency of *gp102* mutations, we concluded that our observed phenotype is attributable to *gp102* loss-of-function.

Many mutations were isolated more than once. In such cases, we have noted which mutants can confidently be considered genetically independent. To determine the frequency at which these mutations arose, we picked single plaques of phage K from a lawn of ATCC 19685 into a small volume of buffer, then diluted and plated the suspended plaques on both the permissive and non-permissive hosts at 37 °C. Each plaque pick contained between 1 × 10^6^ and 1 × 10^7^ PFU. The efficiency of plating of parental phage K was slightly, but statistically significantly, lower on LAC than on 19685 (−0.40 log_10_PFU/mL, *p* = 0.003, *n* = 4). The relative frequency of *gp102* loss-of-function mutations causing clear plaques on the four LAC∆*pot* deletions was thus calculated to be 1.7 × 10^−4^.

### 3.2. In Vitro Antibacterial Phenotypes of Phage K gp102 Mutants

Because the various GHT and GHTP mutants were isolated on different bacterial strains, we used dilution spot tests to screen for any obvious differences in their host ranges. Regardless of which *pot* gene deletion strain was used to isolate each mutant, all GHTP mutants had equivalent efficiencies of plating on the LAC parental strain and on each of LACΔ*potA*, LACΔ*potB*, LACΔ*potC*, and LACΔ*potD*. Similarly, GHT-2 and GHTP-5D1 had equivalent host ranges and efficiencies of plating on 76 distinct strains of laboratory and clinical *S. aureus* and six strains of non-*aureus* staphylococci species.

We selected a subset of the *gp102* mutants to characterize in more detail (Table 1). These mutants were selected to represent a range of effects on gene expression while all being isolated from the same parental lysate. They include deletion of a consensus predicted −35 promoter region, alteration of the ribosome binding site (RBS) (changing AAGGAG to AAAGAG), a nonsense mutation that is expected to yield a 52-amino acid protein instead of a 147-amino acid protein, and two different missense mutations that occur in a similar location as the nonsense mutation. The two missense mutations were each independently isolated at least five times, suggesting that this region is important for protein activity.

Wild-type K and these five representative *gp102* loss-of-function mutants were diluted and spotted on three categories of *S. aureus* strains: permissive to phage K growth at 37 °C (ATCC 19685), less permissive (LAC), and least permissive (LAC∆*potA*, LAC∆*potB*, LAC∆*potC*, LAC∆*potD*). For simplicity, only two *gp102* mutants and one of the LAC∆*pot* strains are shown in Figure 1A, but results were the same for all tested combinations. In keeping with our original observation, wild-type phage K formed clear plaques on the permissive host (ATCC 19685) regardless of temperature (Figure 1A, blue boxes). On LAC, wild-type phage K plaques were smaller and fainter when incubated at 37 °C or when any of the *pot* genes were deleted (Figure 1A, red boxes). The effects of temperature and *pot* gene deletions were additive, to the point where no plaques were visible when wild-type K was plated on LAC∆*pot* strains at 37 °C (Figure 1A, gold box). In contrast, all the *gp102* loss-of-function mutants had the same plaque phenotype when grown under both the most and least permissive of these conditions.

Plaque morphology is not the only measure of phage activity, and it does not always correlate with results obtained in broth cultures [22]. Therefore, the ability of K and the five *gp102* mutants in Table 1 to suppress bacterial growth in broth was also assessed using 24 h growth curves in 96-well plates (Figure 1B). Bacterial growth was inferred from optical density at 600 nm. When incubated at 30 °C, wild-type K and all of the *gp102* mutants completely suppressed the growth of the permissive (ATCC 19685) and non-permissive (LAC and its derivatives) strains. At 37 °C, each phage’s ability to suppress bacterial growth in broth was inversely correlated with presumed Gp102 protein activity. The promoter deletion mutant, which presumably produces little or no *gp102* mRNA, provided the best bacterial growth suppression of LAC and its derivatives. The RBS mutant and ochre mutants, which presumably produce very little protein or a less functional protein fragment, allowed slightly more growth than the promoter deletion mutant. The two substitution mutants, which are expected to produce mutated full-length proteins, allowed more initial bacterial growth but also eventually reduced the density of LAC and its derivatives. In contrast, wild-type phage K provided only partial control of LAC and had no apparent effect on the growth of LAC∆*potA*, LAC∆*potB*, LAC∆*potC*, or LAC∆*potD*. As with the plaque assay, the inhibitory effects of *pot* gene deletion and 37 °C incubation on phage activity were additive.

### 3.3. In Vivo Antibacterial Activity of K and Its gp102 Mutants

**Figure 2:**
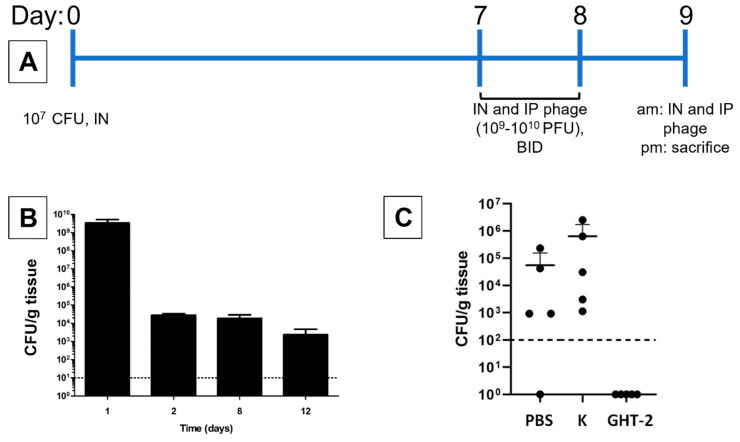
A Gp102 loss-of-function mutation was required for phage K to clear *S. aureus* NRS384 from the mouse upper respiratory tract in a chronic colonization model. (**A**) Timeline of the infection model. (**B**) Bacterial burden in the mouse upper respiratory tract when untreated. (**C**) Effect of treatment with wild-type phage K, the GHT-2 mutant (gp102 RBS is mutated), or the vehicle control (PBS, phosphate-buffered saline). Dotted horizontal lines in panels B and C reflect 10 CFU/g and 100 CFU/g detection limits, respectively.

In preliminary animal experiments, we tested the ability of both K and GHT-2 to decolonize the murine upper respiratory tract (URT) after chronic colonization by USA300 MRSA strain NRS384. In 8-week-old female BALB/c mice, intranasal inoculation with 10^7^ CFU resulted in high levels of nasopharyngeal *S. aureus* 24 h after inoculation, followed by stable colonization at ca. 10^4^ CFU/g of tissue between two and twelve days post-inoculation, which was the last time point tested (Figure 2B). Mice treated with phage K by simultaneous intranasal and intraperitoneal administration had URT bacterial loads equivalent to mice treated with PBS. In contrast, mice treated with phage GHT-2 did not have detectable bacteria in the nasopharynx (limit of detection 1 × 10^2^ CFU/g) (Figure 2C). The absence of detectable phage activity on plates and the equivalent bacterial counts in the PBS and phage K treatment groups indicate that these bacterial counts reflect actual populations in the mice and have not been affected by ex vivo phage activity in the collected samples. The experiment directly comparing phage K and GHT-2 was conducted twice, with similar results.

### 3.4. Correlation of gp102-Mediated Temperature-Sensitivity with S. aureus Lineage

We originally observed temperature sensitive growth of phage K on two USA300 *S. aureus* strains: LAC and NRS384. In order to understand whether this was a coincidence or a true correlation, we reviewed previously collected host range data in which GHTP-5D1 and its wild-type parent (K5), along with GHT-2, were screened for plaque formation at 37 °C on more than 70 unique *S. aureus* strains. There were 51 strains on which both wild-type K and the *gp102* mutants formed plaques. All of the strains on which wild-type K formed much smaller, fainter plaques than the *gp102* mutants were USA300 strains (Table S2). All of the strains on which there was no difference in plaque morphology between wild-type K and the *gp102* mutants were not USA300. There was one non-USA300 strain on which the reverse occurred, with the *gp102* mutants forming smaller fainter plaques than wild-type K. On any given *S. aureus* strain, however, the efficiencies of plating for wild-type K and the *gp102* mutants were similar (within 0.5 log_10_[PFU/mL] for 48 of 51 strains), regardless of plaque morphology.

**Figure 3:**
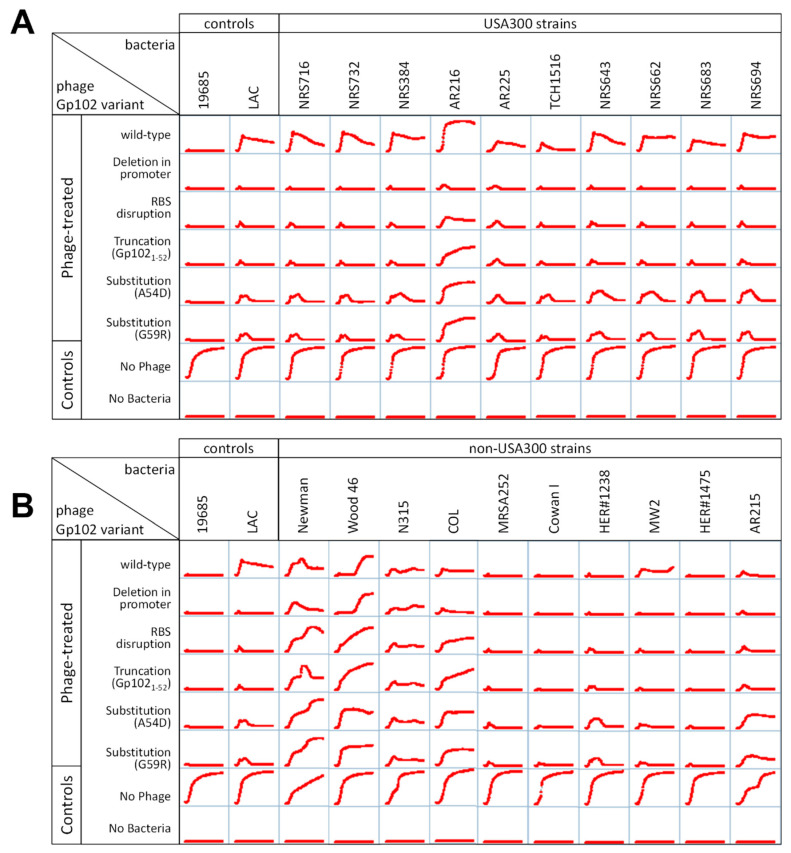
Effect of phage K *gp102* mutants on growth of additional *S. aureus* strains in broth. (**A**) USA300 strains at 37 °C. (**B**) Non-USA300 strains at 37 °C.

To confirm that this correlation held for both the plaquing phenotype and phage activity in broth, we selected ten each of the USA300 and non-300 strains from Table S2 and generated 24 h growth curves in the presence of K and the five representative *gp102* mutants from Table 1. Similar to Figure 1, phage activity was affected by both *S. aureus* lineage and *gp102* sequence at 37 °C (Figure 3) but not at 30 °C. At 37 °C, phage K failed to fully suppress growth of USA300 strains and increasing disruption of the Gp102 protein led to increasing growth suppression (Figure 3A). Among the non-USA300 strains, the ability of phage K to suppress bacterial growth varied by strain, but for 9 of 10 strains, that antibacterial activity was not improved by any of the *gp102* loss-of-function mutations (Figure 3B). The one non-USA300 strain in which *gp102* mutations did improve growth suppression was MW2, which is a USA400 strain that, like USA300, is PVL+ and SCCmec type IV. It is unknown whether these specific factors are causally related to the *gp102*-associated phenotype.

### 3.5. Effect of gp102 Mutations in Other Kayvirus Phages

**Figure 4:**
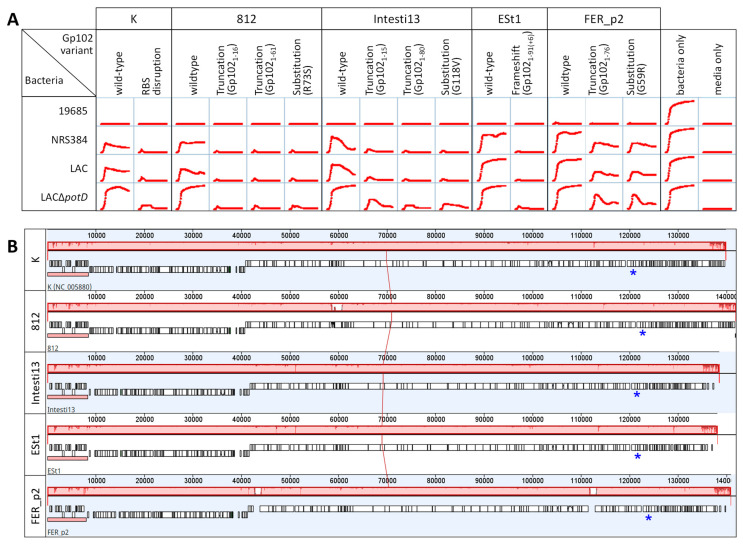
Four *Kayvirus* phages that have *gp102* homologs identical to K show the same temperature-sensitive plaquing and growth suppression phenotypes on USA300 strains. As with K, this phenotype could be suppressed by loss-of-function mutations in the *gp102* homolog. (**A**) Effect of these *gp102* mutations on phage activity in broth at 37 °C. Gp102_1-91(+6)_ indicates a frameshift in codon 92 that creates a downstream stop codon; the protein therefore consists of the first 91 amino acids of Gp102 plus 6 others not usually encoded. (**B**) Intergenomic similarity of the single-copy genomes of parent phages from A, using Mauve. The large blocks of pink indicate nucleotide-level identity among all five phages; red dips and large white gaps indicate regions where the phage genomes differ. Genomes from top to bottom are K (NC_005880), 812 (MH844528), Intesti13, ESt1, and FER_p2. To aid visual comparison, gene annotations were transferred from K onto Intesti13, ESt1, and FER_p2 if there was at least 70% nucleotide similarity. The *gp102* homologs are marked by the blue asterisk.

The nucleotide sequence of *gp102* and its homologs is highly conserved within the *Kayvirus* genus (Figure S1). We selected four phages having genes identical to *gp102* of phage K. We easily isolated *gp102* mutants by plating those phages on LAC∆*potD* at 37 °C (our least permissive condition) at a dilution 4 orders of magnitude more concentrated than the dilution that gave well-isolated plaques on that phage’s regular growth host. Up to three large, clear plaques were picked from those plates and serially passaged twice more on LAC∆*potD* at 37 °C to ensure isolation of clonally pure samples. We amplified and sequenced *gp102* and its flanking regions using the same primers as were used for the GHT and GHTP mutants of K. Most are predicted to be truncation mutants, with one substitution mutant (G59R) recapitulating a mutation that we isolated multiple times in phage K (Figure 4A). The genomes of the four parent phages are very similar (Figure 4B). As with phage K, apparent loss-of-function mutations in *gp102* of these four phages led to better growth suppression of non-permissive strains relative to the wild-type parent phages (Figure 4A).

### 3.6. Effects of gp102 Mutations on Phage Infection Cycle

Our previous observation that all GHTP mutants had equivalent efficiencies of plating on USA300 and non-USA300 strains, regardless of plaque morphology, suggests that major adsorption defects are not involved. We confirmed this by directly testing adsorption rates for the parental stock of K and GHT-2 on MSSA strain ATCC 19685 and on USA300 strain NRS384. There were no differences in adsorption rate based on either phage type or host strain. However, it is possible that later stages of the phage lytic cycle are affected, since both burst size and lysis timing are expected to affect plaque size [23].

**Figure 5:**
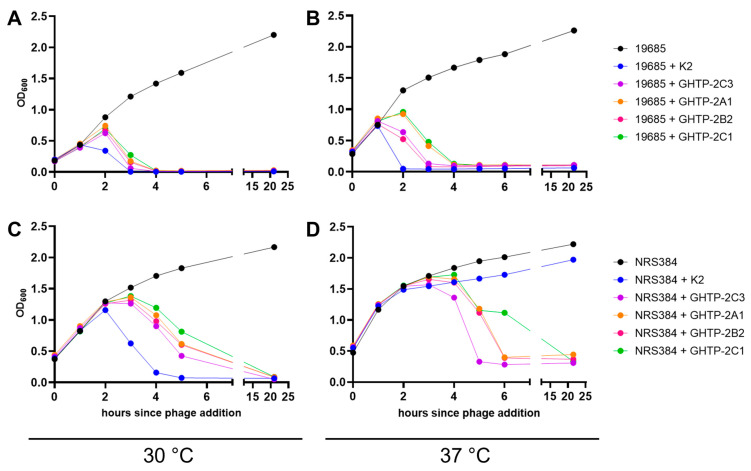
Culture lysis times for phage K with wild-type or mutant *gp102*, under permissive (**A**–**C**) and non-permissive (**D**) infection conditions. (**A**) ATCC 19685 growing at 30 °C, (**B**) ATCC 19685 growing at 37 °C, (**C**) NRS384 growing at 30 °C, and (**D**) NRS384 growing at 37 °C. These data are representative of two replicate experiments.

In larger broth cultures, when the same two *S. aureus* strains were allowed to reach approximately 1 × 10^8^ CFU/mL before phage addition, phage K with wild-type *gp102* (K2) reduced the density of *S. aureus* faster than any of the GHTP mutants under the three permissive conditions tested (Figure 5A–C). The *gp102* loss-of-function mutations were only an advantage on the USA300 strain growing at 37 °C (Figure 5D). This detail was not detectable in the 96-well growth curve experiments because the starting concentration of bacteria at the time of phage addition was too low for OD_600_ measurements to capture changes in cell density. This highlights the importance of studying phage-host interactions using multiple starting conditions.

### 3.7. Gene Expression

**Figure 6:**
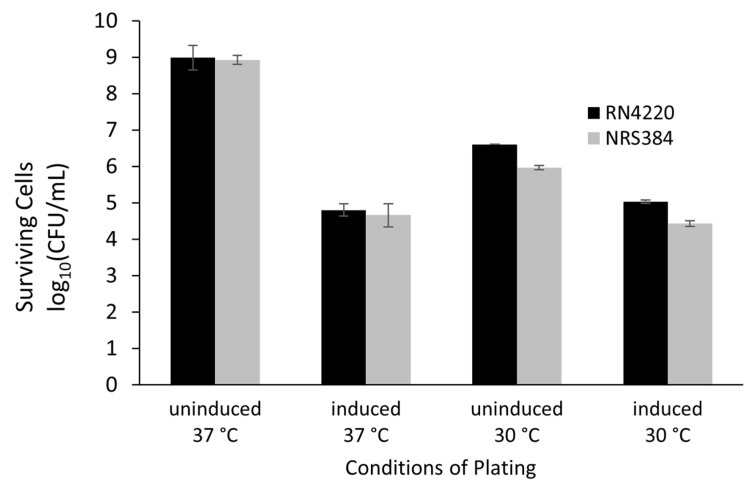
Surviving *S. aureus* cells when a 16 h broth culture of transformed cells is dilution plated at two temperatures in the presence or absence of arsenite, used to induce expression of phage K *gp102*. Values are the mean and standard deviation of log_10_-transformed data from three transformant colonies of each strain.

When expressed under the control of an arsenite-inducible promoter from a plasmid in either *S. aureus* RN4220 (MSSA) or NRS384 (USA300 MRSA), Gp102 reduced cell counts by 4 orders of magnitude at 37 °C (Figure 6). Toxicity was also observed at 30 °C. The small apparent difference in cell survival observed between NRS384 and RN4220 at 30 °C was not statistically significant (*p* > 0.50 for a main effect and all possible interaction effects in a three-factor ANOVA). Notably, a toxic effect of Gp102 expression at 30 °C was seen even when the inducer was absent. Since each replicate used the same initial broth culture for all four plating conditions, this is a real reduction in cell counts and suggests that even slightly leaky expression of *gp102* has a toxic effect at this temperature. The empty plasmid vector was not tested in parallel. However, we are confident that the effect is gene-specific because we are in the process of testing all predicted phage K ORFs in this system and very few have exhibited any toxicity.

### 3.8. Molecular Modeling

Based on primary amino acid sequence, Gp102 is predicted to be 17.5 kDa with a pI of 5.94. We undertook in silico molecular modeling to investigate whether structural features of Gp102 might provide clues to its mechanism of action. HHPred, which uses secondary structure predictions as the basis for protein–protein comparisons, yielded hits dominated by helix-turn-helix (HTH) motifs, including proteins annotated as transposases, DNA-binding proteins, and various transcriptional regulators.

**Figure 7:**
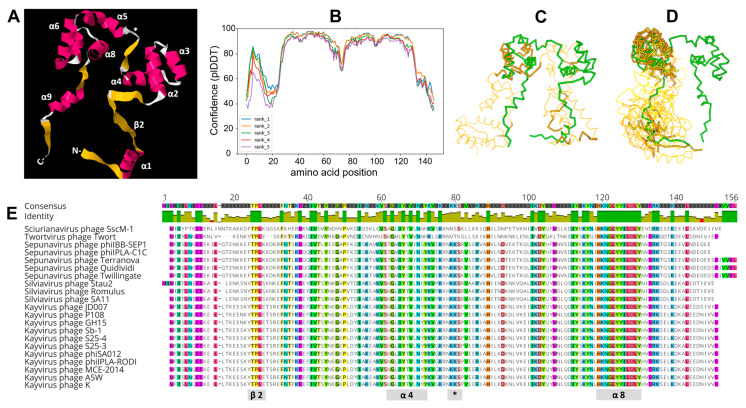
Molecular modeling of Gp102 and structurally similar proteins in other organisms. (**A**) a representative prediction of phage K Gp102 by AlphaFold2, showing pink α-helices (numbered from the N-terminus) and yellow β-strands. (**B**) AlphaFold2 prediction confidence (predicted local distance difference test) by position for the five best predicted structures. The HTH domains are predicted to have high accuracy (pIDDT > 90), regions with plDDT between 70 and 90 have good accuracy, and the terminal “arms” of the protein have low confidence (plDDT < 70) or might only have structure as part of a complex (pLDDT < 50) [CITATION]. (**C**) DALI output showing the superposition of phage K Gp102 (green) and *S. pombe* Reb1 (gold) [Z = 4.5, rmsd = 14.8, lali = 80, %id = 9]. (**D**) DALI output showing superposition of phage K Gp102 (green) and *S. aureus* proteins VraR (RCSB#GVB4), NreC (RCSB#GV7J), a putative DNA-binding response regulator (RCSB#GWVB), and an uncharacterized protein (RCSB#GV5T) [Z = 4.0–4.5, rmsd = 9.3–11.3, lali = 61–63, %id = 13–16]. (**E**) ClustalOmega protein alignment of representative Gp102 homologs across the *Twortvirinae* subfamily, highlighting residues that are conserved in ≥ 90% of the displayed sequences. Selected protein regions are mapped to the secondary structures in panel A. Note that residue numbering is for the aligned consensus sequence and therefore does not match the residue numbering for phage K Gp102 in Table S1.

AlphaFold2 consistently predicted a tertiary structure involving a trihelical (comprising alpha helices 2, 3, 4) and a tetrahelical (alpha helices 5–8) HTH domain connected by a β-strand (Figure 7A). The orientations of the C- and N-terminal regions varied among replicate executions and had very low confidence scores (Figure 7B), which is not surprising given their minimal secondary structure. The two HTH domains span residues Pro32 to Tyr67 and Pro78 to Tyr126, which is where most of the missense mutations captured in our mutant pool were located.

A representative PDB file for Gp102 was used as a query structure in DALI, which uses tertiary structure as the basis for protein-protein comparisons. The only hit in the PDB90 database that had “coverage” of the entire Gp102 protein was *Schizosaccharomyces pombe* protein Reb1 (RCSB#5EYB). *S. pombe* Reb1 is a bifunctional protein that binds specific transcriptional terminators to terminate transcription and to arrest replication forks coming from the opposite direction [24]. The crystal structure of Reb1 complexed with DNA shows the DNA strand passing through the center of the horseshoe-shaped Reb1, with key helices from the two HTH domains making contacts in the major groove [25]. We view these results as hypothesis-generating rather than conclusive. The DALI statistics are relatively weak, and the modest AlphaFold2 confidence in the β-strand regions of Gp102 limits the usefulness of further modeling. However, in the absence of any well-characterized true homologs, the similarity in what seems to be an uncommon overall shape suggests that DNA-binding experiments might be useful for understanding Gp102 function.

Apart from Reb1, the most structurally similar proteins in both the PDB90 and *S. aureus* databases were bacterial transcription factors that contained a single trihelical HTH and that are known to dimerize (e.g., RCSB#GVB4, RCSB#4PCQ). Figure 7 shows superposition diagrams of Gp102 with Reb1 (Figure 7C) and some of the *S. aureus* proteins (Figure 7D).

### 3.9. Taxonomic Distribution of gp102 Homologs

A version of the phage K *gp102* gene is present in all ICTV-recognized species within the *Twortvirinae* subfamily and Figure 7E shows the most conserved amino acid residues. When both ICTV-recognized and unclassified *Twortvirinae* were excluded from blastn results, no other good hits (e-value < 0.001) were found in GenBank or RefSeq records. However, when a similar search was conducted at the protein level (tblastn), proteins with meaningful hits were found throughout the *Herelleviridae* family. The *Herelleviridae* include myophages that infect *Bacillus*, *Enterococcus*, *Listeria*, *Staphylococcus*, and *Lactobacillus* species and that are broadly similar to the *Bacillus* phage SPO1. While taxonomy is not, in itself, phylogeny, the structure of the *Herelleviridae* family is well-supported by a range of genomic and proteomic analyses and correlates well with the phylogenetic relationships among its members [26]. The family, subfamilies, and genera are monophyletic groups, and we use them here as general markers of evolutionary relatedness.

**Figure 8:**
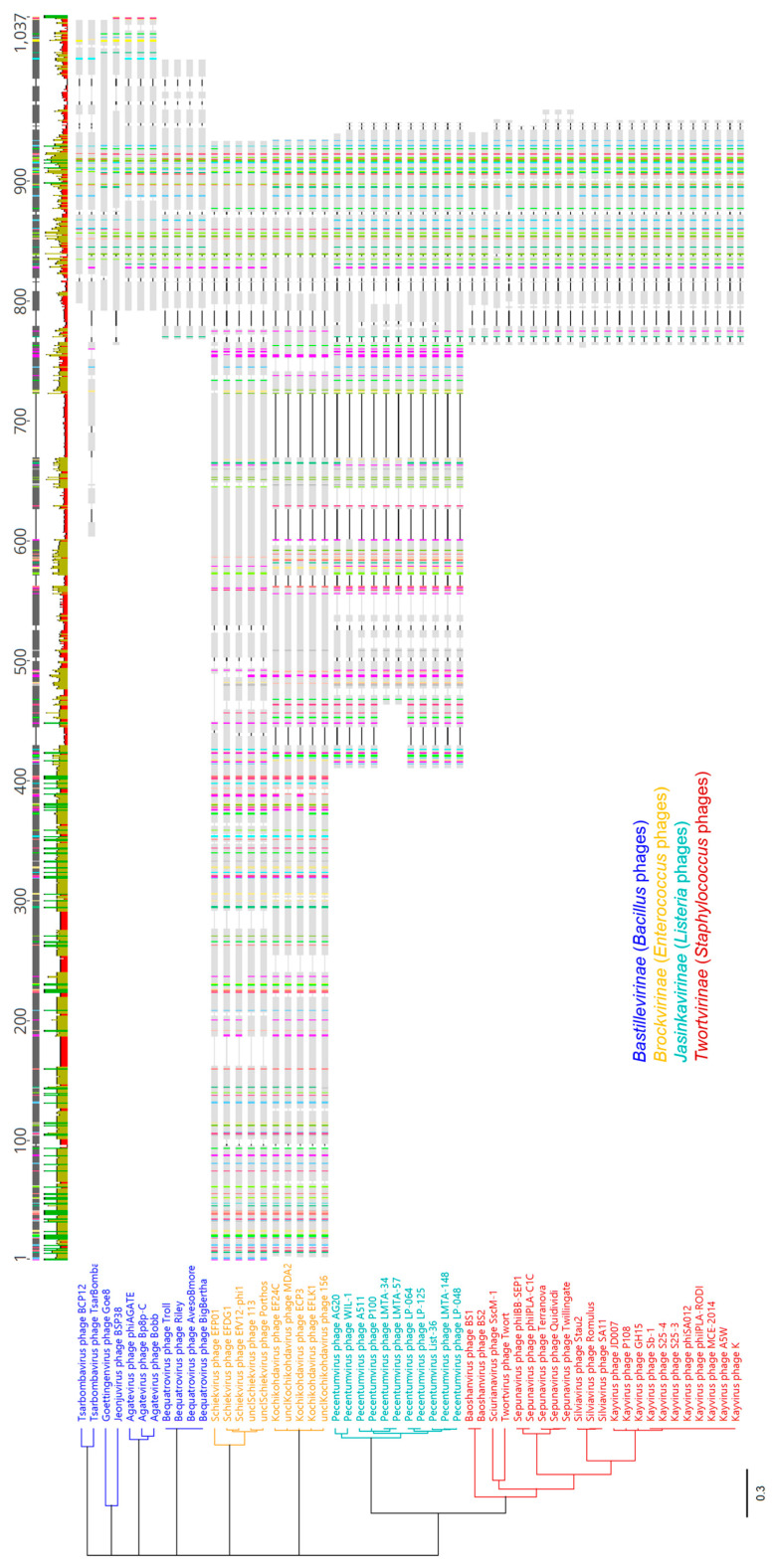
ClustalOmega alignment of predicted proteins with regions similar to Gp102, and a resulting UPGMA tree (Jukes–Cantor model, 100 bootstrap replicates). The alignment includes one representative of each ICTV-recognized species within each genus in which such proteins were found, in addition to two unclassified phages from *Schiekvirus* and *Kochikohdavirus* that were included to increase representation within *Brockvirinae* (sequences listed in Figure S2). All branch points had > 60% consensus support. Above the alignment, gray blocks indicate the majority-rule consensus sequence, and the histogram below shows regions of complete (green), medium (gold), and low (red) percent amino acid identity to the consensus. Colors within each aligned protein indicate residues that are conserved above a 65% threshold.

Proteins containing Gp102-like regions are ubiquitous in the *Twortvirinae* (*Staphylococcus* phages), *Jasinkavirinae* (*Listeria* phages), and *Brockvirinae* (*Enterococcus* phages) subfamilies and entirely absent from the *Spounavirinae* (*Bacillus* phages) subfamily and from the seven genera of *Lactobacillus* phages that did not have subfamily assignments as of ICTV Master Species List #37 (Table S3). Within the *Bastillevirinae*, Gp102 homologs are only identifiable in 5 of 15 genera, but these are the most similar to Gp102 in terms of protein size. Among the *Brockvirinae* and *Jasinkavirinae* phages, there are much larger proteins (approx. 800 and 350 aa, vs. the 147 aa Gp102) that contain a region with apparent homology to Gp102. Figure 8 compares proteins with Gp102-like regions from *Herelleviridae* phages. Protein-level similarity across the Gp102-like region was ≥40% in most cases (Figure S2). None of these proteins have been ascribed functions that are supported by experimental data.

## 4. Discussion

Temperature sensitivity of phage K plaquing has been informally noted by many phage researchers. One hypothesis for this has been that phage K is a laboratory strain that has become attenuated after extended passage with respect to this growth characteristic. After all, other studies have reported consistent plaque formation by relatives of K on various MRSA strains at 37 °C [27,28,29,30], although a few have noted difficulty infecting USA300 strains [31]. Notably however, such studies were typically interested in phage activity at 37° and did not report comparative plaque formation at 30 °C vs. 37 °C. Since plaques do still form at 37 °C when using certain media, and since plaque morphology is expected to be affected by many phage- and host-specific factors, the phenotype might not have been pronounced enough to prompt additional investigation in many circumstances.

It is clear from our genetic data that phage K temperature sensitivity is strongly correlated with the USA300 lineage of *S. aureus* and can be overcome by *gp102* loss-of-function mutations. We demonstrated the same phenomenon in four phages that would be classified as the same species, based on having ≥ 95% nucleotide identity to K. This, coupled with the very high degree of *gp102* nucleotide sequence conservation across the *Kayvirus* genus, points us away from the earlier hypothesis that temperature sensitivity is an issue of phage K attenuation and instead suggests that it is a common phenotype of phages that are closely related to K. In the future, it would be valuable to test whether these findings hold for *Kayvirus* phages that are more distantly related to K (e.g., 70–85% nucleotide-level similarity). When testing our lab’s full phage collection, we did not observe *Silviavirus* phages Romulus and Remus forming smaller, fainter plaques on USA300 vs. non-USA300 strains at 37 °C, but these phages differ from phage K in many other ways that might affect plaque formation.

USA300 strains differ from other *S. aureus* lineages in two main ways. One is the SCCmec type IV cassette, which contains several genes of unknown function and differs substantially from types I through III [32]. It is not clear how this cassette might relate to phage infection processes, but one of our observations suggests a possible association: subtype IVa, present in USA300 strains, is almost identical to subtype IVb, which is present in USA400 strains such as MW2 [33] and MW2 was the only strain on which *gp102* mutants of phage K were better than wild-type at suppressing bacterial growth in broth (although there was no effect on plaque morphology). The second unique feature of USA300 strains is the presence of the arginine catabolic mobile element (ACME), which encodes the *arc* gene cluster (arginine deiminase), the *opp*-3 gene cluster (oligopeptide permease), and *speG* (polyamine acetyltransferase). The *arc* and *speG* genes are both involved in polyamine metabolism, which calls to mind our observations with *S. aureus* LAC, in which the additive effects of temperature and *pot* gene deletions on reducing phage K activity were seen in both plaque assay and broth culture assays. We are conducting separate studies to better understand why deleting components of the polyamine uptake system would have this effect. Polyamines have been shown to play a role in DNA compaction and capsid packaging in some *Escherichia coli*, *Salmonella*, and *Pseudomonas aeruginosa* phages [8,34,35,36]. Since *S. aureus* does not appear to have the genes necessary for synthesis of polyamines de novo [37], impaired uptake could potentially reduce the efficiency of DNA packaging, reducing burst size and therefore plaque size. USA300 MRSA strains additionally reduce the concentration of intracellular polyamines by acetylating them. However, other cations can displace polyamines in the capsids of stored phages [34,38], which suggests that other cations can substitute for polyamines even if polyamines would typically be used for phage K DNA packaging.

As yet, we do not understand the specific function of *gp102*. Direct measurement and efficiency of plating comparisons indicate that *gp102* loss-of-function does not affect phage adsorption. RNA-Seq data from phage K infections of ATCC 19685 show that *gp102* transcription begins almost immediately upon infection, with transcript abundance increasing through 10 min, then dropping back to initial levels by 30 min after phage addition (Kongari et al., manuscript in preparation). These observations are consistent with Gp102 having a role, whether directly or indirectly, in host takeover or lysis timing, though other explanations are also possible especially since protein abundance may not mirror transcript abundance. Additional transcriptional profiling experiments to examine the effects of Gp102 loss-of-function with different host and phage backgrounds is expected to shed light on some of these questions.

Molecular modeling, particularly the similarity of the AlphaFold2 structural predication to *S. pombe* Reb1, suggests that we should investigate the possibility that Gp102 might be a DNA-binding protein with a role in transcriptional regulation. Under this hypothesis, the extremely well-conserved amino acid sequence of the predicted eighth α-helix in *Twortvirinae* Gp102 homologs would be equivalent to the Reb1 helix that fits into the major groove of DNA in the crystal structure of Reb1 complexed with DNA and presumably contributes to Reb1’s binding specificity. We plan to conduct DNA-binding experiments with purified Gp102 in the future as a direct test of Gp102’s DNA binding activity. DALI, which we used to identify the high-level structural similarity of Gp102 and Reb1, might be useful for functional characterization of phage ORFans because it looks for tertiary structure similarities without regard to primary amino acid sequence. ORFans share no obvious sequence similarity with other proteins, but some might have similar structural properties by virtue of having similar functions, such as those involved in early host takeover or transcriptional regulation.

Ultimately, we remain unsure of the role that *gp102* plays in the K-like phages. The very high degree of *gp102* sequence conservation among the *Kayvirus* phages suggests that there is active selection to maintain the integrity of this protein even though its presence is a detriment to phage activity on USA300 MRSA at 37 °C. In a limited strain set, we showed that phage K with wild-type *gp102* kills *S. aureus* more rapidly under permissive conditions, which is likely to give the phage an ecological advantage. The loss-of-function mutations provide an advantage only on USA300 MRSA at 37 °C, when the wild-type phage kills poorly. Since *S. aureus* is an animal-associated organism that can cause serious infections, it typically exists as a skin commensal. Therefore, obligately lytic phages might be most likely to interact with *S. aureus* at temperatures lower than the core body temperatures of their human and animal hosts. This would explain a lack of strong selection for a mutation that increases phage activity on a single lineage at these higher temperatures at the expense of rapid lysis under other conditions. It is also possible that *gp102* loss-of-function might have other negative consequences for phage K. We have not yet tested phage activity against biofilms or in nutrient-limited conditions more representative of the natural environment of *S. aureus*. Under some of these conditions, our GHT and GHTP mutants might have a disadvantage at either 30 °C or even 37 °C.

## Figures and Tables

**Table 1:** Phage K *gp102* mutants used for detailed phenotypic characterization.

Phage K Isolate	Genetic Change ^1^		Predicted Effect on Gp102
	Location in Genome (Location Relative to ORF)	CDS Change	
K2 ^2^	120766…121209 (1…444)	None (WT)	None (WT)
GHTP-2C3	120721 120732…(−45…−33)	-TTA**TG**A**TATAGT** (deletion in promoter)	Impaired transcription
GHTP-2A1	120753 (−13)	G → A (RBS disruption)	Impaired translation
GHTP-2B2	120922 (157)	G → T (ochre mutation)	Truncation after amino acid 52
GHTP-2C1	120926 (161)	C → A	A54D
GHTP-2D1	120940 (175)	G → A	G59R

^1^ All nucleotide and codon numbers are based on NC_005880. For GHTP-2C3, bold text indicates the −10 and extended −10 sequences in the deleted region of the *gp102* promoter. ^2^ K2 is the parental plaque pick from which all of the *gp102* mutants in this table were derived. The sequence of *gp102* in K2 matches that in NC_005880 and is the wild-type (WT).

## Data Availability

Most of the data presented in this study are presented in their entirety in the paper and supplementary material. Other data are being prepared for publication or are available upon request.

## Supplementary Materials

The following supporting information can be downloaded at: https://www.mdpi.com/article/10.3390/v15010017/s1, Table S1: Nature and location of all GHT and GHTP mutants of phage K, relative to the wildtype *gp102* gene; Table S2: Genetic Characterization of *S. aureus* Strains on which both wild-type phage K and *gp102* mutants form plaques; Figure S1: Nucleotide-level similarity (% identity) of the coding sequences for homologs of the phage K *gp102* gene; Table S3: Taxonomic distribution of predicted proteins containing domains similar to Gp102 of phage K. Figure S2. Similarity of the Gp102-like domain of proteins found in *Herelleviridae* phages.
